# Supplementary material for: Exposure to 3′Sialyllactose-Poor Milk during Lactation Impairs Cognitive Capabilities in Adulthood
Source: Nutrients. 2021 Nov 23;13(12):4191. doi: 10.3390/nu13124191 (PMC8707534; doi:10.3390/nu13124191)
Supplement: Supplementary file 1 [file nutrients-13-04191-s001.zip › nutrients-1460566-supplementary.pdf]

Supplementary Materials

Barnes maze

All subjects reached the criterion during the acquisition phase, locating the target hole within the cut-off time. The probe trial, conducted 24 hours after the last acquisition session, revealed that limited access to 3’SL (either during lactation or due to lack of *St3gal4* gene) resulted in a memory impairment. Thus, compared to CTRL, MILK, GENE, and GENE+MILK mice spent less time in the target zone of the apparatus (offspring genotype: $F_{1,41} = 12.43$, $p = 0.001$; maternal genotype: $F_{1,41} = 1.73$, $p = 0.19$; interaction between offspring genotype and maternal genotype: $F_{1,41} = 5.78$, $p = 0.02$; $p < 0.05$ in post-hoc tests, see Supplementary fig. 1a).

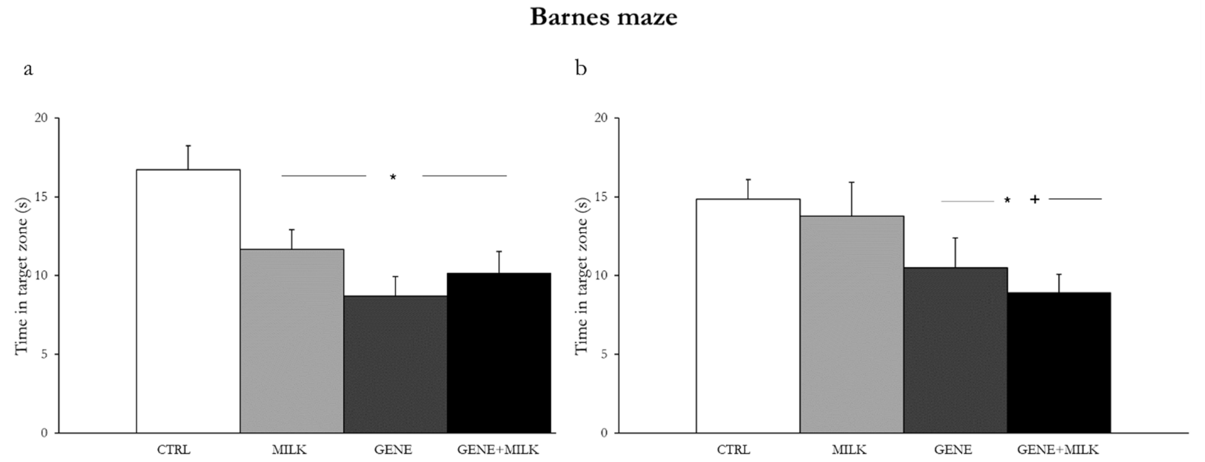

Supplementary figure S1. Time spent in the target zone of the Barnes maze during the test performed: a) 24 hours after the last acquisition trial and b) one week after the last acquisition trial. * $p < 0.05$ in post-hoc tests compared to CTRL group. + $p < 0.05$ in post-hoc tests compared to MILK group.

During the probe trials conducted one week after last acquisition session, the memory retention varied depending on offspring genotype (offspring genotype: $F_{1,41} = 7.19$, $p = 0.01$; maternal genotype: $F_{1,41} = 0.58$, $p = 0.45$; interaction between offspring genotype and maternal genotype: $F_{1,41} = 0.02$, $p = 0.89$). Specifically, GENE and GENE+MILK groups spent less time in the target zone compared to CTRL and MILK group ($p < 0.05$ in post-hoc tests, see Supplementary fig. 1b).

Attentional set-shifting task

In accordance with the general phenotype observed in the ASST, the total number of trials and errors during all the phases of the attentional set-shifting task varied depending on the specific stage of the task (stage: $F_{4,152} = 7.53$, p = <0.0001 for trials; stage: $F_{4,152} = 11.86$, p = <0.0001 for errors). Specifically, CDr has represented the most challenging stage for the experimental animals, with more trials attained to complete the criterion and more errors compared to all the other stages ($p < 0.05$ in post-hoc tests). Additionally, in accordance with our predictions, reduced access to 3'SL during lactation resulted in deficits in attention in terms of trials (maternal genotype: $F_{1,38} = 11.37$, p = 0.002) and errors (maternal genotype: $F_{1,38} = 10.4$, p = 0.003). Additionally, we observed that this phenotype was partially moderated by the offspring phenotype (trials: offspring genotype x maternal genotype: $F_{1,38} = 7.73$, p = 0.0084; errors: offspring genotype x maternal genotype: $F_{1,38} = 7.22$, p = 0.01). Specifically, while MILK mice required a higher number of trials and committed more errors than CTRL groups ($p < 0.05$ in post-hoc tests), such difference was not present in KO offspring, with the exclusion of the EDs. The difference between MILK and CTRL was consistent across all stages of the task with the exclusion of the EDs (see Supplementary fig. 2).

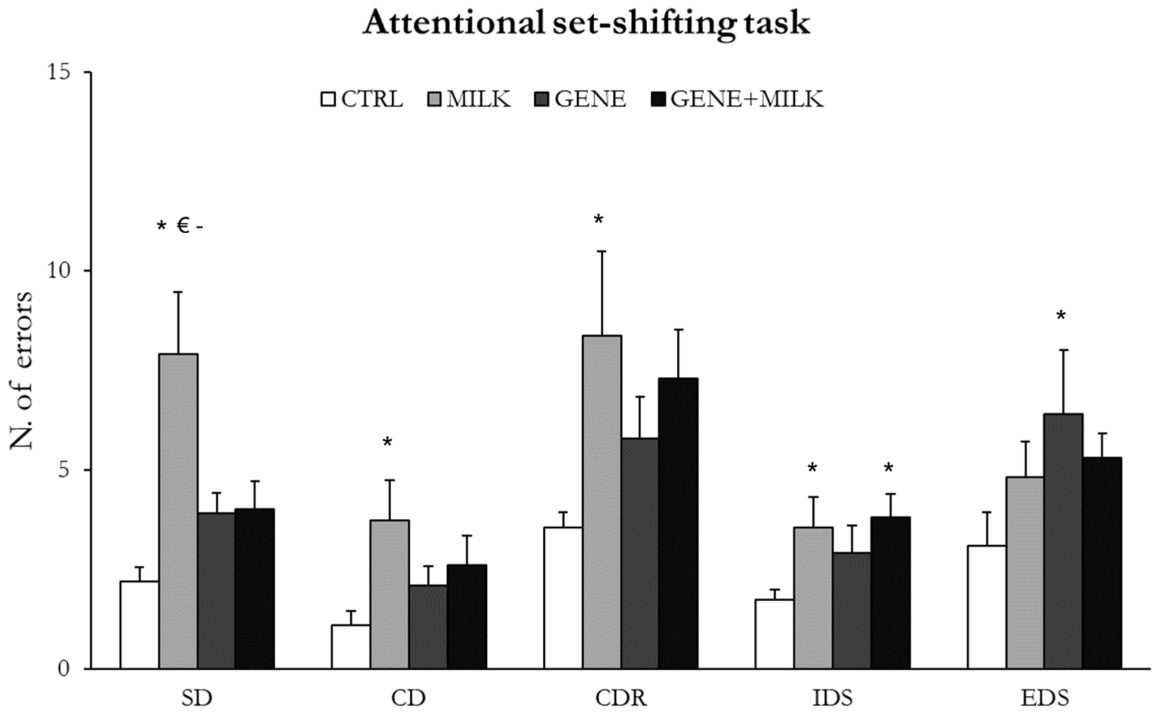

Supplementary figure S2. Number of errors to attain the criterion of each single phase of the tests (8 correct trials out of 10). * $p < 0.05$ in post-hoc tests compared to CTRL group. € $p < 0.05$ in post-hoc tests compared to GENE group. – $p < 0.05$

in post-hoc tests compared to GENE + MILK group. SD: simple discrimination, CD: compound discrimination, CDR: compound discrimination reversal, IDS: intra-dimensional shift, EDS: extra-dimensional shift.

Glucose tolerance test

After glucose administration, all groups exhibited a rapid increase in blood glucose concentration, attaining maximal values 20 minutes following administration. Then, blood glucose concentration declined to reach baseline values 120 minutes after the injection (time: $F_{4,168} = 61.83$, $p < 0.0001$). We observed that a constitutive absence of 3'SL resulted in a reduced response to glucose administration (offspring genotype: $F_{1,42} = 14.063$, $p = 0.0005$) whereby GENE and GENE+MILK mice exhibited lower blood glucose concentration both compared with CTRL and MILK mice ($p < 0.05$ in post-hoc tests), see Supplementary fig.3. Apparently, these differences were independent on the rearing dam (maternal genotype: $F_{1,42}= 0.58$, $p = 0.46$; interaction between offspring genotype × maternal genotype: $F_{1,42} = 0.55$, $p = 0.47$).

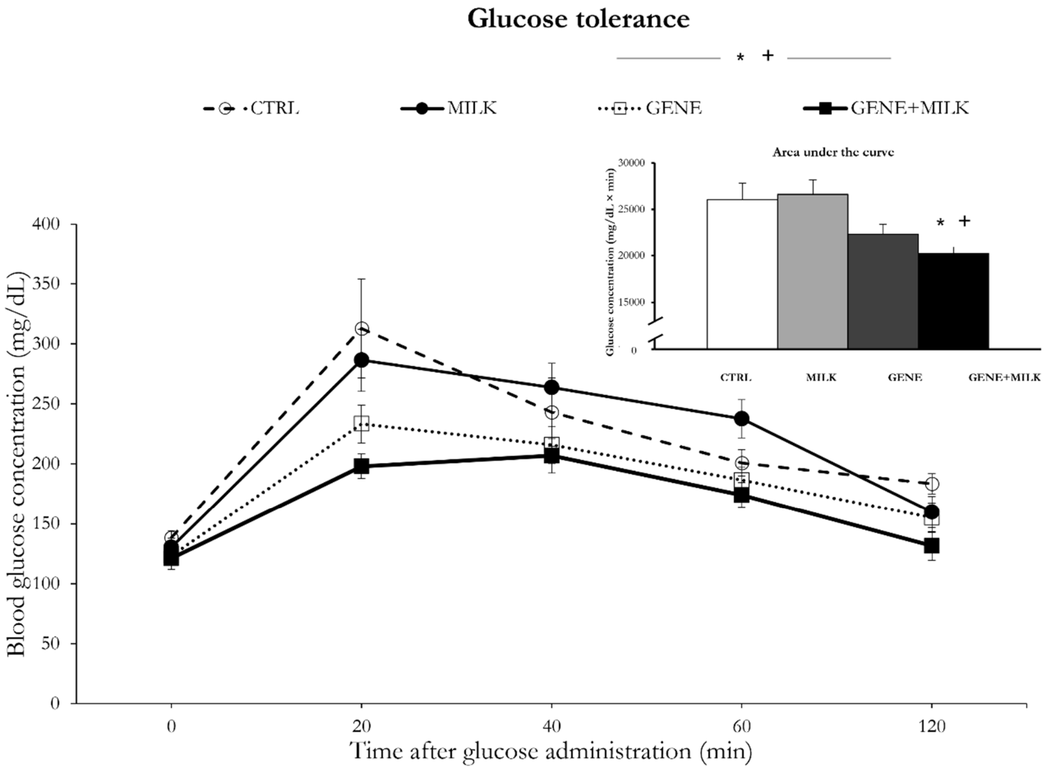

Supplementary figure S3. Blood glucose concentration after IP glucose administration. Mice with constitutive absence of 3'SL result in a reduced physiological response to glucose, independently from the mother's genotype. Inset: integral response to glucose administration plotted as the area under the curve. * $p < 0.05$ in post-hoc tests compared to CTRL group, + $p < 0.05$ in post-hoc tests compared to MILK group.

T-maze

In accordance with expectations, CTRL subjects exhibited a natural tendency to alternate between the two arms of the maze (95 % CI, 67.66 to 83.96). Yet, such natural tendency was modulated by the maternal genotype (maternal genotype: $F_{1,41} = 4.86$, $p = 0.03$) whereby MILK mice exhibited reduced spontaneous alternation compared to CTRL ($p < 0.05$ in post-hoc tests, see Supplementary fig. 4). KO mice did not differ from CTRL subjects regardless of the foster dam (offspring genotype: $F_{1,41} = 0.46$, $p = 0.5$; interaction between offspring genotype and maternal genotype: $F_{1,41} = 2.08$, $p = 0.16$).

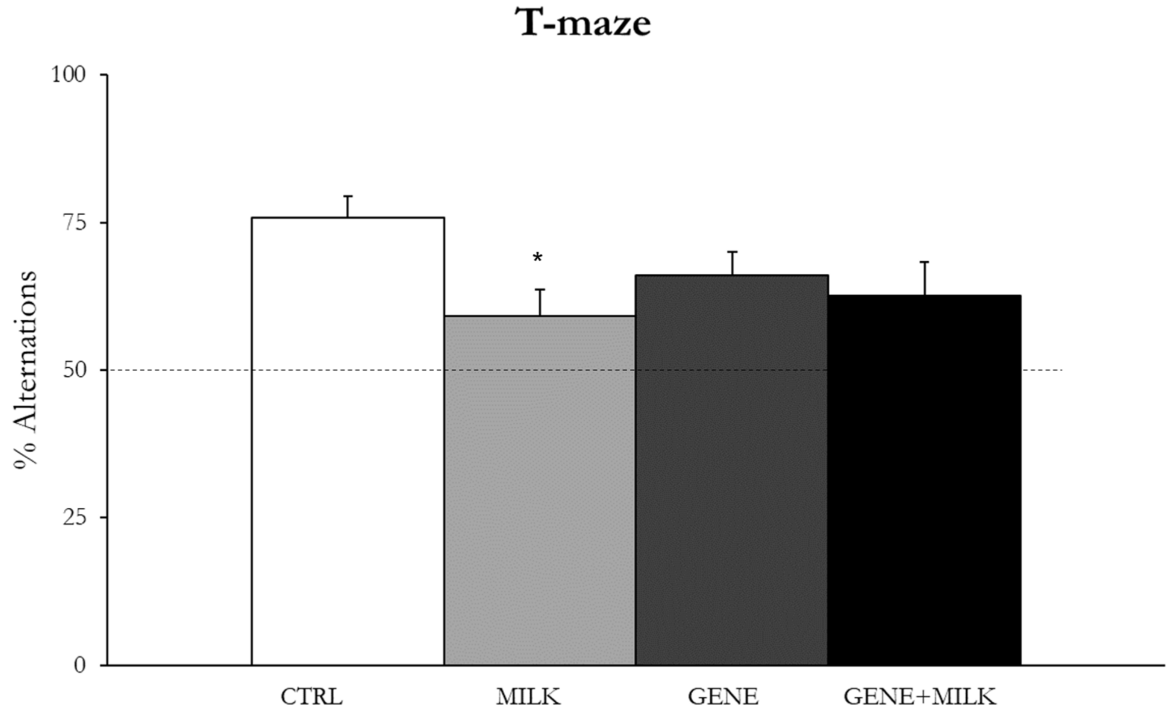

Supplementary figure S4. Percent of alternations exhibited by subjects of the four experimental groups. MILK mice (WT offspring reared to KO dams) exhibited a reduced number of spontaneous alternations compared to CTRL. * $p < 0.05$ in post-hoc tests compared to CTRL group. Dashed line represents the chance level.

Elevated 0-maze

All subjects showed a preference for the closed sector compared to the open sector (T-test, t-value = 44.55, $p < 0.0001$). Such preference varied between experimental groups as a function of the offspring genotype (offspring genotype: $F_{1,41} = 4.66$, $p = 0.04$; maternal

genotype: $F_{1,41} = 1.33$, $p = 0.25$; interaction between offspring genotype and maternal genotype: $F_{1,41} = 0.008$, $p = 0.93$). Specifically, GENE mice exhibited higher preference for the closed sector compared to MILK group ($p < 0.05$ in post-hoc tests).

Pre-pulse inhibition

On average, all subjects showed an intact PPI whereby they all exhibited a reduced startle in response to prepulse plus pulse trials compared to prepulse alone trials (95 % CI, 11.51 to 30.99). Furthermore, this capability did not vary between experimental groups (offspring genotype: $F_{1,43} = 1.64$, $p = 0.29$; maternal genotype: $F_{1,43} = 0.22$, $p = 0.64$; interaction between offspring genotype and maternal genotype: $F_{1,43} = 0.96$, $p = 0.33$).

Sucrose preference

All subjects exhibited a strong preference for the sucrose solution. This preference did not vary between experimental groups (offspring genotype: $F_{1,43} = 4.77*10^{-5}$, $p = 0.99$; maternal genotype: $F_{1,43} = 0.083$, $p = 0.77$; interaction between offspring genotype and maternal genotype: $F_{1,43} = 1.07$, $p = 0.31$).

General locomotion

The absence of 3′SL from maternal milk did not influence the locomotory activity. Yet, GENE+MILK group showed lower locomotory activity compared to all other groups, indicating an effect of the pairing between 3′SL poor milk and constitutive absence of *St3Gal4* on the general locomotion (day × hour × offspring genotype: $F_{69,2967} = 2.45$, $p = <0.0001$; day × hour × maternal genotype: $F_{69,2967} = 2.27$, $p = <0.0001$; day × hour × offspring genotype × maternal genotype: $F_{69,2967} = 1.28$, $p = 0.06$; $p < 0.05$ in post-hoc tests), see Supplementary fig.5.

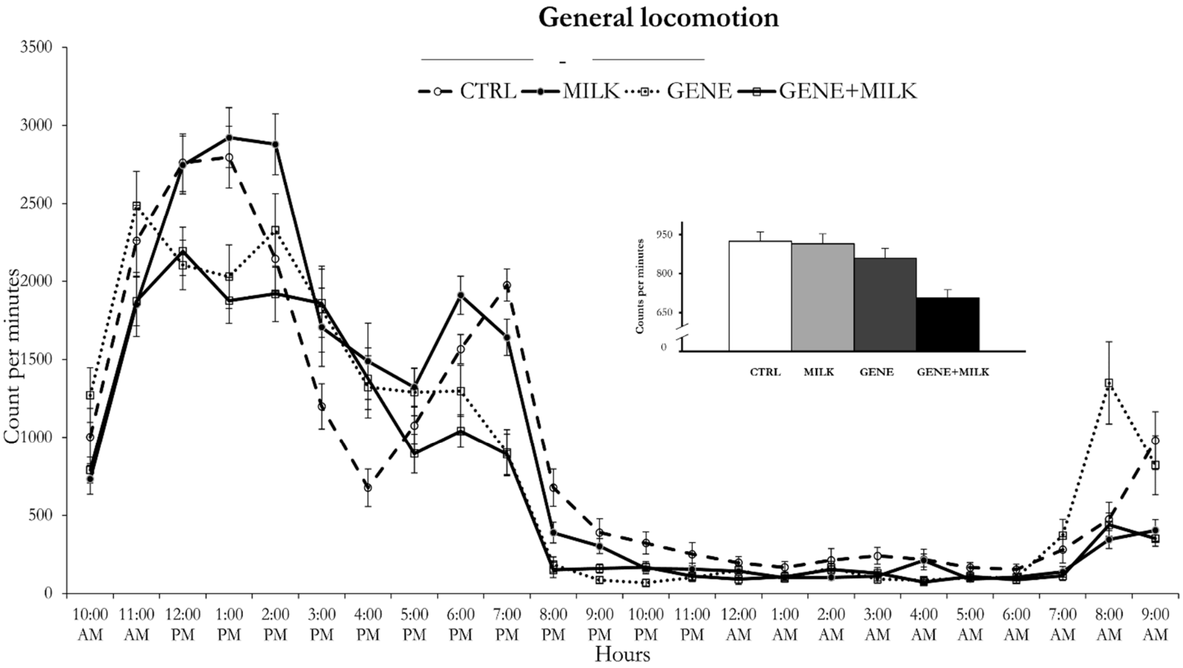

Supplementary figure S5. Locomotor activity (counts per minute) recorded during four consecutive days. Data represented are the average of locomotor activity measured over four consecutive days, 24 hours per day. GENE+MILK exhibited lower total locomotory activity compared to the other groups. Inset: absolute levels of locomotion (counts per minute) irrespective of the diurnal cycle. - $p < 0.05$ in post-hoc tests compared to GENE + MILK group.

Object recognition memory task

During both test phases, conducted one hour and 24 hours after the last session of the habituation phase, all subjects showed a preference for the novel object compared to the familiar one, spending more time with the novel object compared to the old one (for short-term test: T-test, t-value = 4.78, $p < 0.0001$; for long-term test: T-test, t-value = 7.07, $p < 0.0001$). Such preference did not vary between experimental groups in both probe trials (for short-term test: offspring genotype: $F_{1,42} = 1.13$, $p = 0.72$; maternal genotype: $F_{1,42} = 0.01$, $p = 0.91$; interaction between offspring genotype and maternal genotype: $F_{1,42} = 0.11$, $p = 0.74$; for long-term test: offspring genotype: $F_{1,41} = 0.028$, $p = 0.87$; maternal genotype: $F_{1,41} = 0.023$, $p = 0.88$; interaction between offspring genotype and maternal genotype: $F_{1,41} = 0.33$, $p = 0.57$, see Supplementary fig. 6) thus suggesting that all experimental subjects had an intact recognition memory.

Novel object recognition test

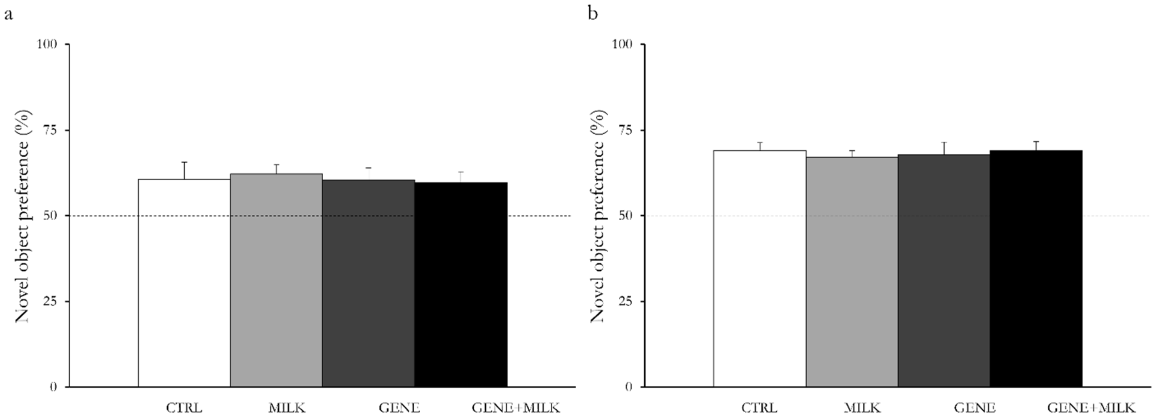

Supplementary figure S6. Percent preference for the novel object, considering the interaction behaviours with the objects. There are no differences between experimental groups. Dashed line represents the chance level.
